# Novel SCN1A mutation in the IFMT motif of the α1 subunit of the voltage-gated NaV1.1 channel causing familial hemiplegic migraine

**DOI:** 10.1186/1129-2377-14-S1-P19

**Published:** 2013-02-21

**Authors:** B De Vries, CM Weller, O De Fàbregues, SC Koelewijn, AH Stam, J Haan, MD Ferrari, GM Terwindt, AMJ van den Maagdenberg

**Affiliations:** 1 Department of Human Genetics, Leiden University Medical Centre (LUMC), Netherlands; 2 Department of Neurology, Corporació Sanitària Parc Taulí, Sabadell, Barcelona, Spain; 3 Department of Neurology, Leiden University Medical Centre (LUMC), Netherlands

## Introduction

Familial hemiplegic migraine (FHM) is a monogenic subtype of migraine with aura. So far three FHM genes are identified; the CACNA1A gene, the ATP1A2 gene and the SCN1A gene.

## Objective

To investigate the genetic cause of FHM in a Spanish four-generation family.

## Methods

We assessed the clinical features in the four affected family members by direct interview (proband and her offspring) and from heteroanamnestic information from the proband about her father. We performed direct sequencing of the FHM genes. After exclusion of mutations in the CACNA1A and ATP1A2 genes, direct sequencing of the SCN1A gene was performed.

## Results

The proband had life-long hemiplegic migraine attacks. At age 69, she had a prolonged episode of hemiplegia, which gradually resolved completely over the course of a month. Her father, one of her three children, and one of her grandsons were also affected by hemiplegic migraine, but with a much lower attack frequency. We identified a novel missense mutation (c.4460G>C; p.Ile1498Met) in exon 24 of the SCN1A gene in all tested FHM patients of the family. The mutation is located in the intracellular loop of the protein and affects the IFMT (4 amino acids) motif, which is essential for inactivation of the encoded NaV1.1 sodium channel.

## Conclusion

p.Ile1498Met is only the sixth SCN1A mutation identified thus far. It is the first mutation in the IFMT motif that causes pure FHM without additional symptoms, albeit with a large variability in severity and frequency of hemiplegic migraine attacks among mutation carriers.

